# Supplementary material for: Diversity, Antimicrobial, Antioxidant, and Anticancer Activity of Culturable Fungal Endophyte Communities in Cordia dichotoma
Source: Molecules. 2023 Oct 4;28(19):6926. doi: 10.3390/molecules28196926 (PMC10574381; doi:10.3390/molecules28196926)
Supplement: Supplementary file 1 [file molecules-28-06926-s001.zip › molecules-2604414-supplementary.pdf]

Diversity, Antimicrobial, Antioxidant, and Anticancer Activity of Culturable Fungal Endophyte Communities in *Cordia dichotoma*

Table S1. Tissue specific diversity of endophytic fungi isolated from *C. dichotoma.*

Tissues	Root	Stem	Leaf	Total
**Segment examined**	180	168	113	461
**Segment infected**	79	47	22	148
**Total isolates**	85	58	26	169
**Endophytic strains**	8	6	5	19
**Total CR%**	43.89%	27.97%	19.46%	
**Total IR%**	47.22%	34.52%	23%	
**Shannon wiener diversity index**	1.992	1.645	1.46	

CR indicate colonization rate. IR indicate isolation rate.

Table S2. Morphological characterization of endophytic fungi.

Fungal code	Colony characteristics on SDA media	Pigmentation	Microscopic appearance	Plausible endophytic fungus
MCR1	initially white / yellowish	Brown	Septate, unbranched conidiophore	*Aspergillus* sp.
MCR2	Appears dark brown/black in colour, cottony, woolly in nature	Brown/Black	Septate, conidiophores appeared alone or in small groups, and were frequently branching, linear, and having bends.	*Alternaria* sp.
MCR3	Appears black in colour	Black	hyaline, thin-walled and aseptate, cylindrical to subovoid in shape	*Lasiodiplodia* sp.
MCR4	Appears white/brown	Yellow	Aseptate, conidiophores, chain of spores formed.	*Talaromyces* sp.
MCR5	Appears brown/white in colour	Brown	ovoid or ellipsoidal, pale brown, smooth-walled conidia	*Alternaria* sp.
MCR6	Appears white in colour	brown	chlamydospores, oval conidia, and round pycnidia	*Epicoccum* sp.
MCR7	Appears white colonies	Brown	Conidia, globular shape	*Aspergillus* sp.
MCR8	Appears black/white in colour	Black	Conidia, septa; conidiophore solitary; branched/ unbranched);	*Alternaria* sp.
MCS1	initially white then black.	Black	Conidia, septate and hyaline hyphae.	*Aspergillus*sp
MCS2	Appears brown/ blackish colour	Brown/Orange	Septate, erect conidiophores, and conidia.	*Cladosporium*sp
MCS3	Appears white in col-	Brown	ovoid or ellipsoidal co-	*Alternaria* sp.

	our		nidia	
MCS4	Appears whitecolour	Brown	flask-shaped or cylindrical phialides	*Aspergillus* sp.
MCS5	Appears white/brown in colour	Brown	Round and ovoid	*Oligoporus* sp.
MCS6	Appears white/yellow in color, slimy texture	White	Aseptate, presence of conidiophores and phialides	*Talaromyces* sp.

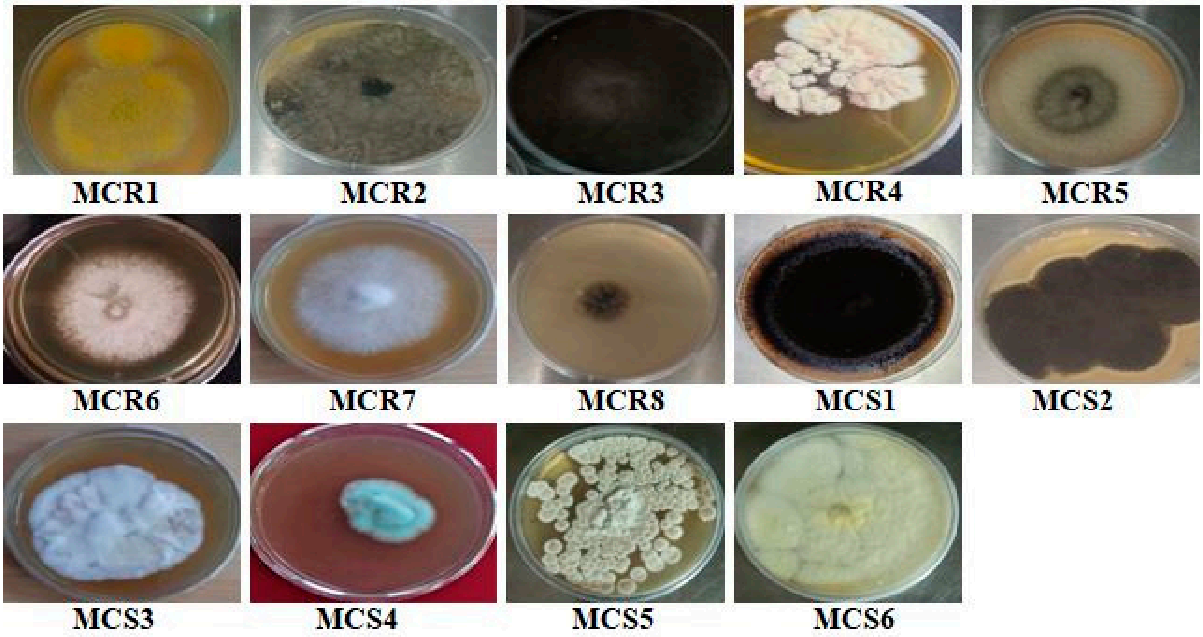

Figure S1. Morphological identification of isolated fungal endophytes sourced from *C. dichotoma.*

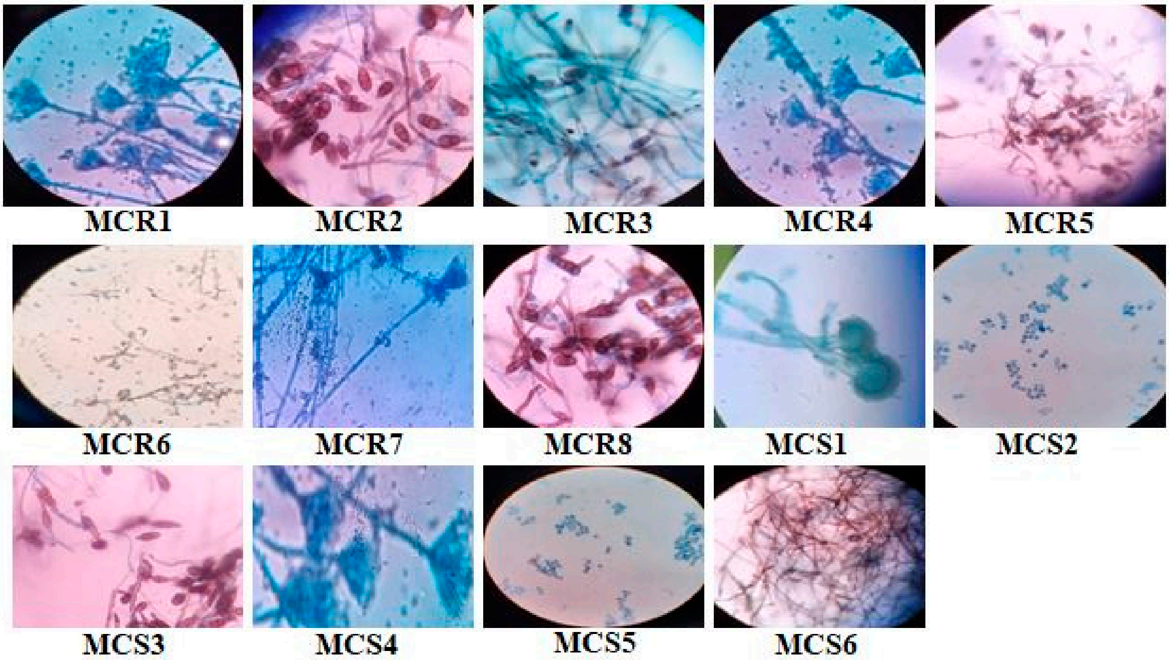

Figure S2. Microscopical identification of isolated fungal endophytes sourced from *C. dichotoma.*

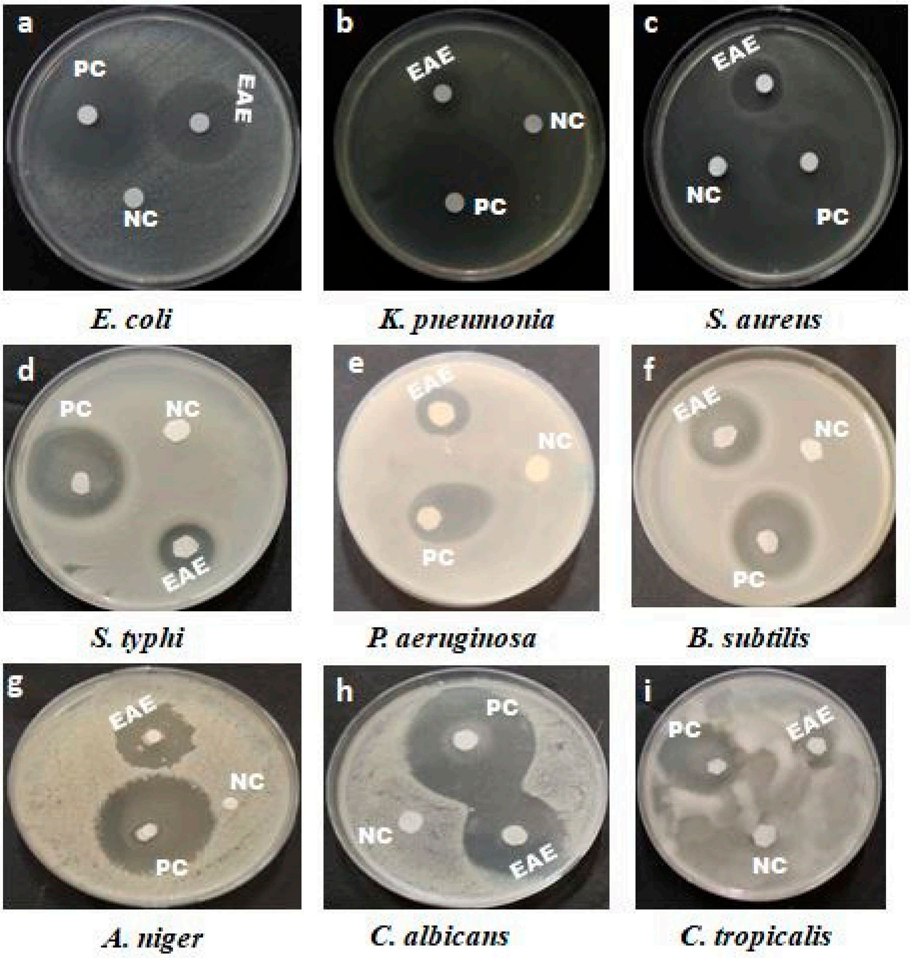

Figure S3. The zone of inhibition (mm) of ethyl acetate crude extract (EAE) of *Alternaria destruens* (MCR2) at a concentration of 1 mg/mL against (a) *E. coli* (b) *K. pneumonia* (c) *S. aureus* (d) *S. typhi* (e) *P. aeruginosa* (f) *B. subtilis* (g) *A. niger* (h) *C. albicans* (i) *C. tropicalis*. PC: Chloramphenicol and Fluconazole at concentration of 1 mg/mL serve as positive control; NC: DMSO used as negative control.

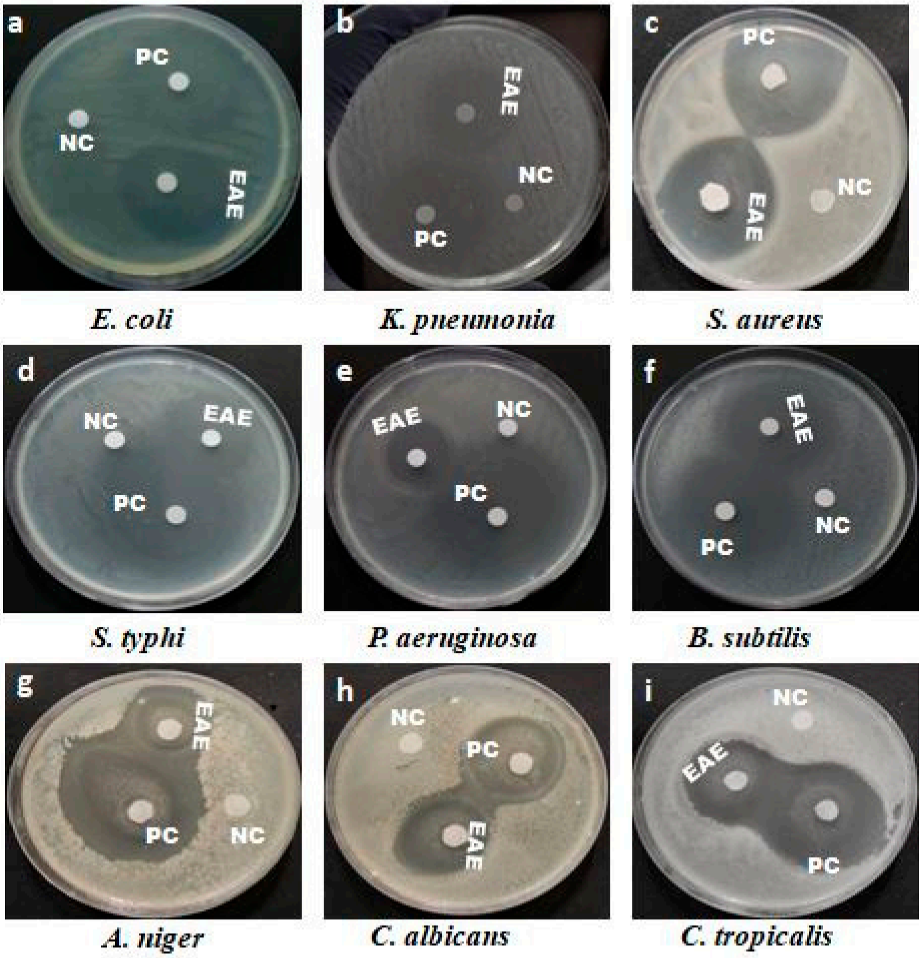

Figure S4. The zone of inhibition (mm) of ethyl acetate crude extract (EAE) of *Lasiodiplodiatheobromae* (MCR3) at a concentration of 1 mg/mL against (a) *E. coli* (b) *K. pneumonia* (c) *S. aureus* (d) *S. typhi* (e) *P. aeruginosa* (f) *B. subtilis* (g) *A. niger* (h) *C. albicans* (i) *C. tropicalis*. PC: Chloramphenicol and Fluconazole at concentration of 1 mg/mL serve as positive control; NC: DMSO used as negative control.

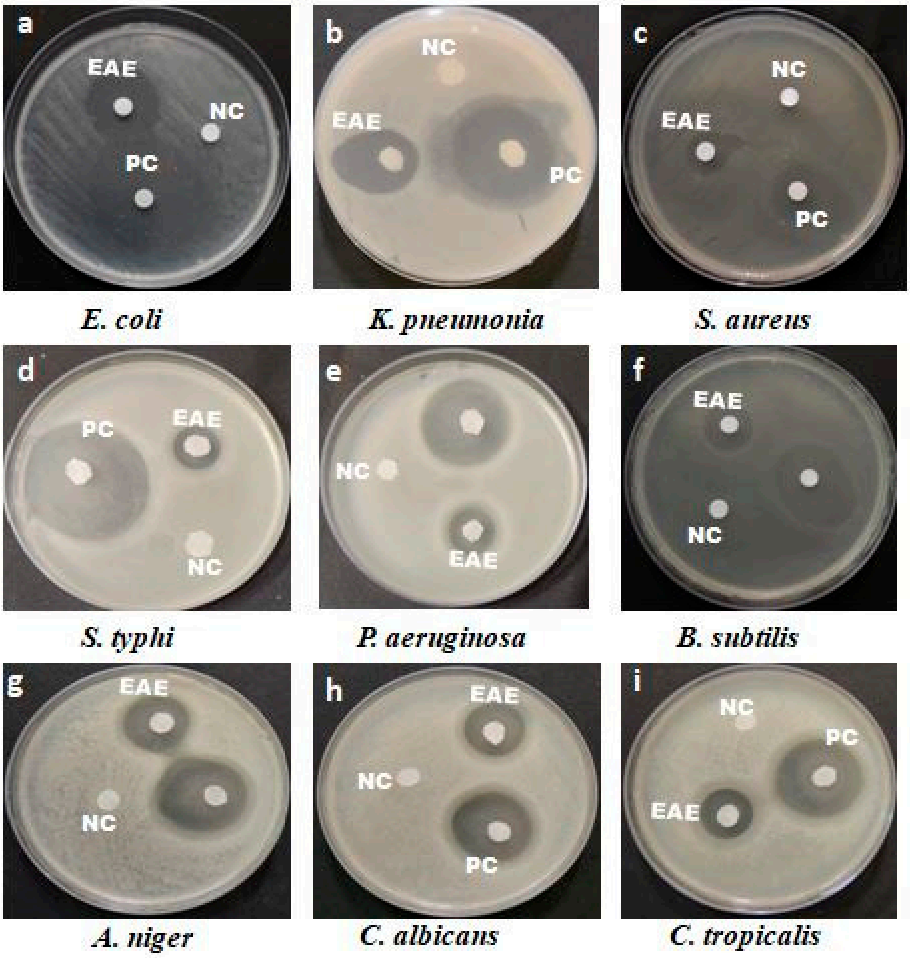

Figure S5. The zone of inhibition (mm) of ethyl acetate crude extract (EAE) of *Alternaria angustiovoidea* (MCR8) at a concentration of 1 mg/mL against (a) *E. coli* (b) *K. pneumonia* (c) *S. aureus* (d) *S. typhi* (e) *P. aeruginosa* (f) *B. subtilis* (g) *A. niger* (h) *C. albicans* (i) *C. tropicalis.* PC: Chloramphenicol and Fluconazole at concentration of 1 mg/mL serve as positive control; NC: DMSO used as negative control.

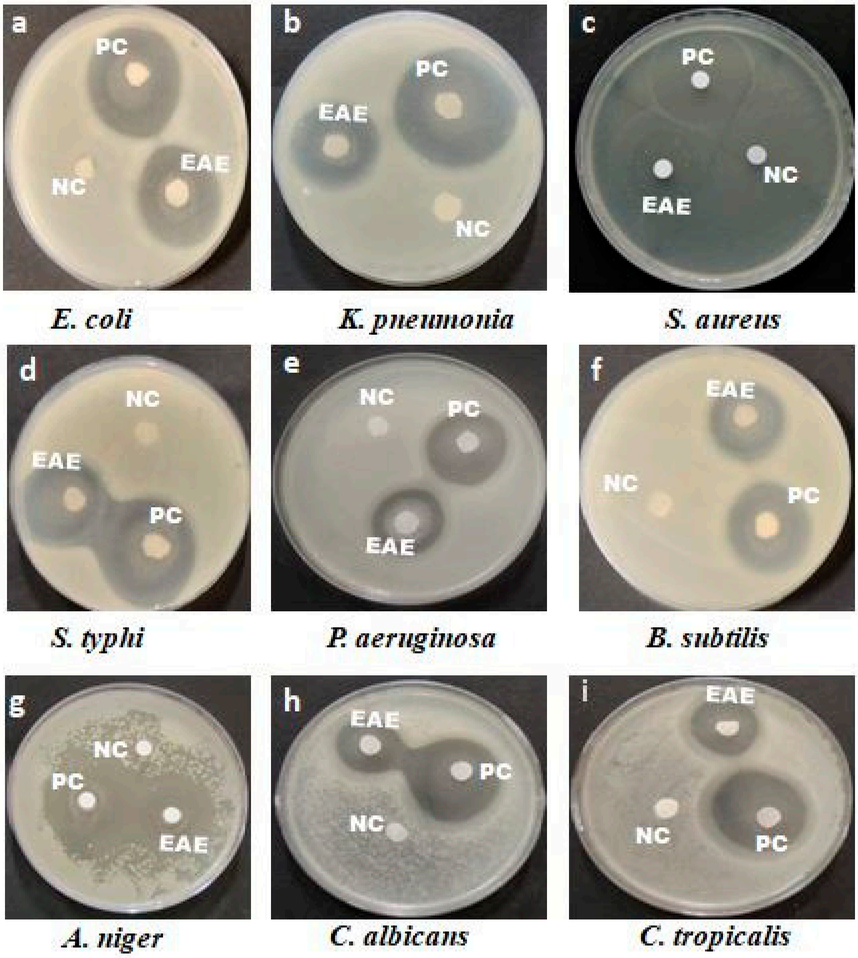

Figure S6. The zone of inhibition (mm) of ethyl acetate crude extract (EAE) of *Cladosporium cladosporioides* (MCS2) at a concentration of 1 mg/mL against (a) *E. coli* (b) *K. pneumonia* (c) *S. aureus* (d) *S. typhi* (e) *P. aeruginosa* (f) *B. subtilis* (g) *A. niger* (h) *C. albicans* (i) *C. tropicalis*. PC: Chloramphenicol and Fluconazole at concentration of 1 mg/mL serve as positive control; NC: DMSO used as negative control.

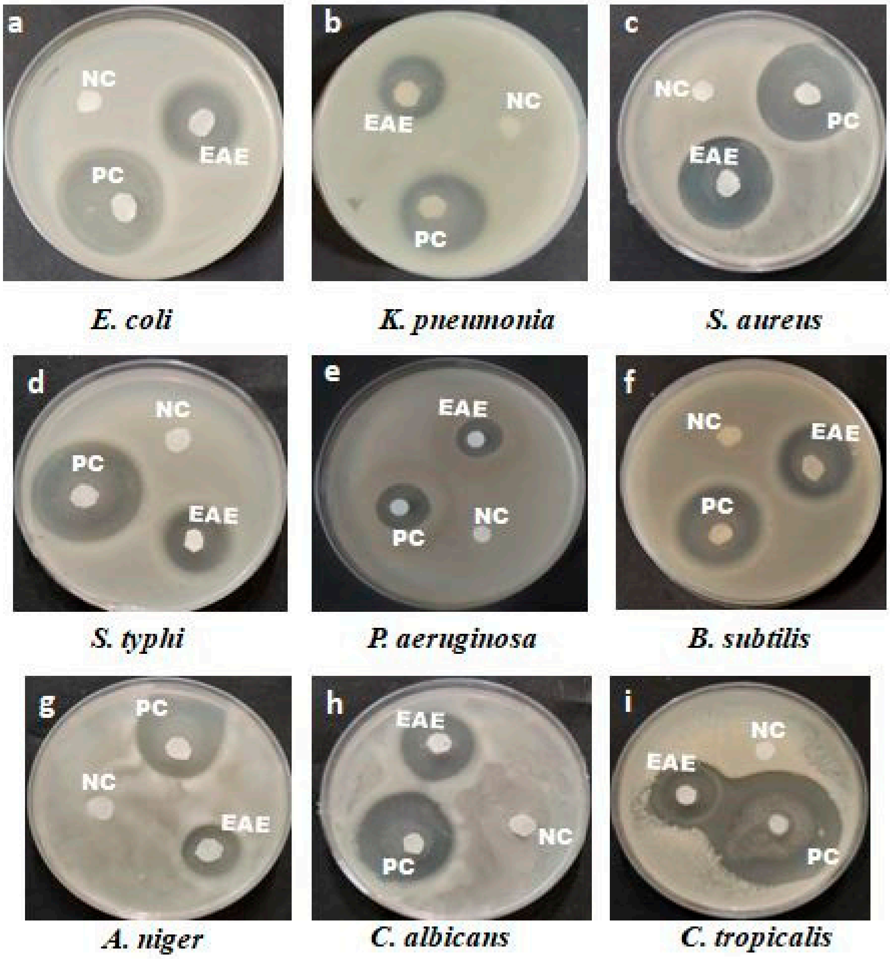

Figure S7. The zone of inhibition (mm) of ethyl acetate crude extract (EAE) of *Talaromyces purpureogenus* (MCS6) at a concentration of 1 mg/mL against (a) *E. coli* (b) *K. pneumonia* (c) *S. aureus* (d) *S. typhi* (e) *P. aeruginosa* (f) *B. subtilis* (g) *A. niger* (h) *C. albicans* (i) *C. tropicalis.* PC: Chloramphenicol and Fluconazole at concentration of 1 mg/mL serve as positive control; NC: DMSO used as negative control.

Table S3. Chemical composition of ethyl acetate extract of *Cladosporium cladosporioides*(MCS2) from GCMS analysis.

S.No.	Retention time in min	Compounds	Molecular Formula	Molecular weight g/mol	Peak area%
1	4.22, 4.46	2,3-Butanediol	$C_4H_{10}O_2$	90.121	1.6
2	7.53	1,3-Oxathiane	C_4H_8OS	104.17	0.20
3	9.64	Phenol	C_6H_6O	94.11	0.08
4	10.06	Decane	$C_{10}H_{22}$	142.29	0.19
5	10.39	Benzene, 1,3-dichloro-	$C_6H_4Cl_2$	147	0.10
6	10.84	2-Acetylcyclopentanone	$C_7H_{10}O_2$	126.15	0.19
7	12.13	Lomustine	$C_9H_{16}ClN_3O_2$	233.695	0.36
8	12.90	Phenylethyl Alcohol	$C_8H_{10}O$	122.16	8.67
9	14.56	Azulene	$C_{10}H_8$	128.17	0.21
10	14.82	Dodecane	$C_{12}H_{26}$	170.33	0.82
11	15.53	3-Phenylpropanol	$C_9H_{12}O$	136.19	1.92
12	16.07	Acetic acid, 2-phenylethyl ester	$C_{10}H_{12}O_2$	164.20	0.18
13	16.52, 22.66	Dodecane, 2,6,11-trimethyl-	$C_{15}H_{32}$	212.41	0.42
14	17.45	Nonane, 5-(1-methylpropyl)-	$C_{13}H_{28}$	184.36	0.21

15	18.73	1-Tetradecene	$C_{14}H_{28}$	196.37	0.34
16	18.89	Tetradecane	$C_{14}H_{30}$	198.39	2.70
17	19.52	Benzeneethanol, 4-hydroxy-	$C_8H_{10}O_2$	138.16	1.65
18	21.28	Dodecane, 2-methyl-	$C_{13}H_{28}$	184.36	0.55
19	21.36	Cycloheptasiloxane, tetradecamethyl-	$C_{14}H_{42}O_7Si_7$	519.07	0.24
20	21.79	2,4-Di-tert-butylphenol	$C_{14}H_{22}O$	206.32	0.39
21	23.01	(-)-Mellein	$C_{10}H_{10}O_3$	178.18	0.61
22	24.14	1-Heptadecene	$C_{17}H_{34}$	238.5	0.56
23	24.35	Hexadecane	C16H34	226.41	3.05
24	25.24	Benzophenone	$C_{13}H_{10}O$	182.21	0.22
25	26.12	Cyclooctasiloxane, hexadecamethyl-	$C_{16}H_{48}O_8Si_8$	593.2	0.15
26	27.02	Tetradecane, 4-methyl-	$C_{15}H_{32}$	212.41	0.34
27	28.10	Phenol, pentachloro-	C_6HCl_5O	266.33	0.38
28	28.26	Hexadecane, 1-iodo-	$C_{16}H_{33}I$	352.34	0.18
29	28.68, 32.10, 34.95	1-Nonadecene	$C_{19}H_{38}$	266.5	1.9
30	28.81, 32.19	Heneicosane	$C_{21}H_{44}$	296.57	3.21
31	28.98	Tridecane, 3-cyclohexyl-	$C_{19}H_{38}$	266.5	0.19
32	29.75	Tributyrin	$C_{15}H_{26}O_6$	302.36	0.28
33	30.60, 34.66	Tetrapentacontane	$C_{54}H_{110}$	759.4	0.82
34	30.76	Tricosyl acetate	$C_{25}H_{50}O_2$	382.7	0.20
35	31.03	Silane, trichlorooctadecyl-	$C_{18}H_{39}Cl_3Si_2$	387.9	0.50
36	31.13	Pyrrolo[1,2-a]pyrazine-1,4-dione, hexahydro-3-(2-methylpropyl	$C_{11}H_{18}N_2O_2$	210.27	0.46
37	31.61	Cyclohexanone, 2,3,3-trimethyl-2-(3-methylbutyl)-	$C_{14}H_{26}O$	210.36	7.57
38	31.68	Dibutyl phthalate	$C_{16}H_{22}O_4$	278.34	11.46
39	31.87	2,2-Dimethyl-3,6-dioxa-9-thia-2-silahenicosane	$C_8H_{16}O$	128.21	0.37
40	31.96, 37.83	Cyclononasiloxane, octadecamethyl-	$C_{18}H_{54}O_9Si$	667.4	2.13
41	32.42	Cyclobutanecarboxylic acid, tridecyl ester	$C_{18}H_{30}O_2$	278.4	0.20
42	32.72	Oleyl alcohol, trifluoroacetate	$C_{20}H_{35}F_3O_2$	364.5	0.24
43	33.13	Cyclohexanol, 3-(aminomethyl)-3,5,5-trimethyl-	$C_{10}H_{21}NO$	171.28	1.23
44	33.47	Acetic acid, chloro-, octadecyl ester	$C_{20}H_{39}ClO_2$	347	0.20
45	33.56	3-Decanynoic acid	$C_{10}H_{18}O_3$	186.25	2.93
46	34.04	11-Methyltricosane	$C_{24}H_{50}$	338.7	0.21
47	34.21	9-Methylheneicosane	$C_{22}H_{46}$	310.60	0.58
48	34.53	Octadecanoic acid	$C_{18}H_{34}O_2$	282.5	0.93
49	34.80	2,5-Piperazinedione, 3,6-bis(2-methylpropyl)-	$C_{12}H_{22}N_2O_2$	226.32	3.56
50	35.02	Eicosane	$C_{20}H_{42}$	282.5	1.06
51	35.17	Dotriacontyl propyl ether	$C_{32}H_{66}$	450.9	0.26
52	35.30	3-Cyclohexylnonadecane	$C_{25}H_{50}$	350.6	0.26
53	35.41	(Z)-9-octadecen-4-olide	$C_{18}H_{32}O_2$	280.4	0.33
54	36.34	i-Propyl 5,9,17-hexacosatrienoate	$C_{29}H_{52}O_2$	432.7	1.28
55	36.50	Methyl 5,9-octadecadienoate	$C_{19}H_{34}O_2$	294.5	0.21

56	37.51	Spiculesporic acid	$C_{17}H_{28}O_6$	328.4	23.21
57	37.66	Pyrrolo[1,2-a]pyrazine-1,4-dione, hexahydro-3-(phenylmethyl	$C_{14}H_{16}N_2O_2$	244.29	4.60
58	37.99	Propanoic acid, 2-methyl-, 1,7,7-trimethylbicyclo[2.2.1]hept-2-yl	$C_{14}H_{24}O_2$	224.33	0.23
59	38.73, 40.12	Dotriacontane	$C_{32}H_{66}$	450.88	0.44
60	39.48	Bis(2-ethylhexyl) phthalate	$C_{24}H_{38}O_4$	390.55	1.83
61	40.85	L-Prolinamide, 5-oxo-L-prolyl-L-phenylalanyl-4-hydroxy-	$C_{19}H_{24}N_4O_4$	372.4	0.61

Table S4. Metabolite profiling of *C. cladosporioides* (MCS2) by LCMS analysis.

S.No	Compound	RT	Molecular formula, mass g/mol	m/z	Activity	References
1	L-Serine O-sulfate	1.32	$C_3H_7NO_6S$ 185.16	184.966 3	Antimicrobial, Antioxidant	[1]
2	Se-Methyl-L-selenocysteine	1.36	$C_4H_9NO_2Se$ 182.09	182.964 4	Anticancer	[2]
3	3,5-Dibromo-L-tyrosine	1.79	$C_9H_9Br_2NO_3$ 338.98	336.926 9	Antioxidant, Anticancer	[3]
4	Quintozene	1.86	$C_6C_{l5}NO_2$ 295.3	292.846 1	Antimicrobial, Anticancer	[4]
5	Doxorubicin	2.23	$C_{27}H_{29}NO_{11}$ 543.51	543.134 4	Anticancer	[5]
6	Estramustine	4.49	$C_{23}H_{31}C_{l2}NO_3$ 440.40	439.144 6	Antioxidant, Anticancer	[6]
7	10-Formyldihydrofolate	4.85	$C_{20}H_{21}N_7O_7$ 471.4	471.115 5	Anticancer	[6]
8	Disulfiram	6.32	$C_{10}H_{20}N_2S_4$ 296.53	296.072 3	Anticancer	[7]
9	Prunasin	6.51	$C_{14}H_{17}NO_6$ 295.29	295.081 5	Anticancer, Antioxidant	[8]
10	Xylitol	7.52	$C_5H_{12}O_5$ 152.15	152.072 2	Anticancer	[9]
11	Dodecanedioic acid	11.45	$C_{12}H_{22}O_4$ 230.30	230.141 3	Anticancer	[10]
12	6-endo-Hydroxycineole	13.26	$C_{10}H_{18}O_2$ 170.252	170.121	Antioxidant, Anticancer	[10]
13	5,10-Methylenetetrahydro-folate	16.72	$C_{20}H_{23}N_7O_6$ 457.44	457.132 3	Anticancer, Antimicrobial	[11]
14	Linustatin	21.17	$C_{16}H_{27}NO_{11}$ 409.39	409.185 1	Anticancer	[12]
15	Delphinidin	31.93	$C_{15}H_{11}O_7$ 303.24	303.054 9	Antioxidant, Anticancer	[13]

16	Se-Adenosylselenomethionine	33.85	$C_{15}H_{23}N_6O_5Se$ 446.4	447.097	Antibacterial, Anticancer	[14]
17	Naringenin	39.8415	$C_{15}H_{12}O_5$ 272.25	272.0656	Antimicrobial, Anticancer	[15]

Bibliography

1. Kim, S.; Kim, J.; Kim, N.; Lee, D.; Lee, H.; Lee, D.; Kim, K. Metabolomic elucidation of the effect of sucrose on the secondary metabolite profiles in *Melissa officinalis* by ultraperformance liquid chromatography-mass spectrometry. *ACS Omega.* 2020, **5**(51), 33186-33195. https://doi.org/10.1021/acsomega.0c04745.
2. Mondal, A.; Banerjee, S.; Bose, S.; Mazumder, S.; Haber, R.; Farzaei, M.; Bishayee, A. Garlic constituents for cancer prevention and therapy: From phytochemistry to novel formulations. *Pharmacol.Res.* 2022,175, 105837. https://doi.org/10.1016/j.phrs.2021.105837.
3. Youssef, D.; L.A. Shaala. Psammaplysins: Insights from Natural Sources, Structural Variations, and Pharmacological Properties. *Marine Drugs.* 2022, 20(11), 663. https:// doi.org/10.3390/md20110663.
4. Silva, N.; Umsza, M.; Ramos, D.; Gálvez, J.; Paula, T.; Balderrama, A. Comparison of the biological potential and chemical composition of Brazilian and Mexican propolis. *Applied Sciences.* 2021, 11(23), 11417. https://doi.org/10.3390/app112311417.
5. Rocha, P.; Campos, J.; Nunes, V.; Vieira, M.; Boleti, A.; Rabelo, L.; Dos, E.; Picoli, K. Antioxidant and protective effects of *Schinus terebinthifoliu Raddi* against doxorubicin-induced toxicity.*Appl.Biochem.Biotechnol.* 2018, 184, 869-884. https://doi.org/10.1007/s12010-017-2589-y.
6. Tafrihi, M.; Imran, M.; Tufail, T.; Gondal, T.; Caruso, G.; Sharma, S.; Sharma, R.; Atanassova, M.; Atanassov, L.; Valere, Patrick. The wonderful activities of the genus Mentha: Not only antioxidant properties. *Molecules.* 2021,26(4), 1118. https://doi.org/10.3390/molecules26041118.
7. Jiao, Y.; Hannafon, B.; Zhang, R.; Fung, K.; Ding, W. (2017) Docosahexaenoic acid and disulfiram act in concert to kill cancer cells: A mutual enhancement of their anticancer actions. *Oncotarget.* 2021, 8(11), 17908. https://doi.org/10.18632%2Foncotarget.14702.
8. Hussain, S.; Javed, M.; Abid, M.; Khan, M.; Syed, S.; Faizan, M.; Feroz, F. *Prunus Avium* L.; Phytochemistry, Nutritional and Pharmacological Review. *Advancements in Life Sciences.* 2021, 8(4), 307-314.
9. Kim, W.; Choi, W.; Lee, S.; Kim, W.; Lee, D.; Sohn, U.; Shin, H.; Kim, W. Anti-inflammatory, antioxidant and antimicrobial effects of artemisinin extracts from *Artemisia annua* L. *Korean J. Physiol. Pharmacol.* 2015, 9(1), 21.http://dx.doi.org/10.4196/kjpp.2015.19.1.21.
10. Weintraub, S.; Shpigel, T.; Harris, L.; Schuster, R.; Lewis, E.; Lewitus, D. Astaxanthin-based polymers as new antimicrobial compounds.*Polymer Chemistry.* 2017,8(29), 4182-4189. https://doi.org/10.1039/C7PY00663B.
11. Elsharkawy, E.; Ed, A.; Abdallah, E.; Ali, A. The Genus Rhodococcus as a source of novel bioactive substances: A review.*J. Pharmacogn. Phytochem.* 2017, 6(3), 83-92.
12. Raimondi, M.; Randazzo, O.; Franca, M.; Barone, G.; Vignoni, E.; Rossi, D.; Collina, S. DHFR inhibitors: Reading the past for discovering novel anticancer agents. *Molecules.* 2019,24(6), 1140. https://doi.org/10.3390/molecules24061140.
13. Ryu, J.; Kang, H.; Cho, S. Changes over the fermentation period in phenolic compounds and antioxidant and anticancer activities of blueberries fermented by *Lactobacillus plantarum*. *J.Food Sci.* 2019, 84(8), 2347-2356. https://doi.org/10.1111/1750-3841.14731.
14. Hou, W.; Xu, H. Incorporating selenium into heterocycles and natural products-From chemical properties to pharmacological activities.*J.Med.Chem.* 2022,65(6), 4436-4456. https://doi.org/10.1021/acs.jmedchem.1c01859.
15. Kozłowska, J.; Grela, E.; Baczyńska, D.; Grabowiecka, A.; Anioł, M. Novel O-alkyl derivatives of naringenin and their oximes with antimicrobial and anticancer activity. *Molecules.* 2019, 24(4), 679. https://doi.org/10.3390/molecules24040679.
